# Offset Analgesia and Onset Hyperalgesia: A Comparison Between Women With Non‐Suicidal Self‐Injury and Healthy Women

**DOI:** 10.1002/brb3.71659

**Published:** 2026-08-15

**Authors:** Jens Fust, Granit Kastrati, Johan Bjureberg, Nitya Jayaram‐Lindström, Clara Hellner, Eva Kosek, Peter Fransson, Karin B. Jensen, Maria Lalouni

**Affiliations:** ^1^ Department of Clinical Neuroscience Karolinska Institutet Stockholm Sweden; ^2^ Institute for Globally Distributed Open Research and Education (IGDORE) Gothenburg Sweden; ^3^ Department of Clinical Neuroscience Centre for Psychiatry Research, Karolinska Institutet Stockholm Sweden; ^4^ Stockholm Health Care Services, Region Stockholm Stockholm Sweden; ^5^ Department of Surgical Sciences Uppsala University Hospital, Uppsala University Uppsala Sweden

**Keywords:** Nociception, Hyperalgesia, Hypoalgesia, Functional magnetic resonance imaging

## Abstract

**Background:**

Individuals who engage in non‐suicidal self‐injury (NSSI) exhibit reduced pain sensitivity compared to the general population. It has been argued that this hypoalgesic characteristic may be attributable to hyper‐effective pain modulation. However, empirical support for this theory remains inconsistent. The aim of the study was to use a combined offset analgesia (OA) and onset hyperalgesia (OH) protocol to investigate if women with NSSI have a propensity to inhibit nociceptive signals to a higher degree (stronger OA response) and facilitate nociceptive signals to a lesser degree (weaker OH response), compared to a control group.

**Methods:**

Data was collected from 76 women, 18–35 age (37 with NSSI and 39 healthy controls). The OA and OH protocol were combined with functional magnetic resonance imaging (fMRI).

**Results:**

The NSSI group displayed a weaker OH response, compared to the control group. This suggests that women with NSSI do not facilitate nociceptive signals to the same extent as healthy women. However, there were no significant differences between the groups regarding OA. Across all participants, we observed stronger activation in the primary sensory cortex during the OH condition, compared to the control condition.

**Conclusions:**

The results offer partial support for the general hypothesis that women with NSSI demonstrate enhanced pain modulation. The fMRI finding requires replication.

## 1 Introduction

Individuals who engage in non‐suicidal self‐injury (NSSI) tend to be less sensitive to pain than the general population. Not only do many individuals with NSSI report feeling little or no pain while harming themselves (Izadi‐Mazidi et al. 2019; Lloyd‐Richardson et al. 2007; Zetterqvist et al. 2013), but also, in laboratory studies the NSSI population display higher pain threshold, higher pain tolerance, and report lower pain intensity when being exposed to a painful stimulus, compared to control groups (Koenig et al. 2016). Longitudinal data suggest that high pain tolerance is a risk factor for severe NSSI engagement (Boyne and Hamza, 2024).

It has been theorized that this hypoalgesic trait could be explained by hyper‐effective pain modulation (Defrin et al. 2020; Lalouni et al. 2022), that is, efficient pain inhibition (e.g., conditioned pain modulation) combined with low pain facilitation (e.g., temporal summation), consistent with the anti‐nociceptive extreme of the pain modulation continuum as defined by Yarnitsky et al. (2014). Previous studies on pain modulation in participants with NSSI show mixed findings, with some reporting enhanced conditioned pain modulation (Defrin et al., 2020; Lalouni et al. 2022), others reduced conditioned pain modulation (Leone et al., 2021), and no evidence of abnormal temporal summation (Lalouni et al., 2022). This inconsistency makes it difficult to draw strong conclusions about pain modulation in this population. It is also important to note that individuals without pain conditions can show markedly different pain modulation profiles, ranging from strong inhibition to net facilitation of pain (Petersen et al. 2025; Vincenot et al. 2026). Offset analgesia (OA) is an alternative protocol to characterize pain inhibition (Grill and Coghill, 2002). While conditioned pain modulation could be said to measure spatial filtering of nociceptive information, OA is related to temporal filtering of nociceptive information (Nahman‐Averbuch et al. 2014). The OA protocol compares a constant noxious heat stimulation with an identical heat stimulation, apart from a brief period of increased temperature. The short deviation in temperature leads to a disproportionate reduction of pain intensity, compared to the constant heat stimulation. It has also been demonstrated that a hyperalgesic response can be produced using an inverted version of the OA protocol, called onset hyperalgesia (OH) (Alter et al. 2020). Brain imaging studies have found that the OA response is associated with activity in the brainstem (Derbyshire and Osborn, 2009; Nahman‐Averbuch et al. 2014; Yelle et al. 2009; Zhang et al. 2018) and dorsolateral prefrontal cortex (Alter et al. 2022; Nahman‐Averbuch et al. 2014; Yelle et al. 2009; Zhang et al. 2018).

Because of the mixed results in the studies investigating NSSI‐related hypoalgesia, there is a need to further characterize the pain modulation of the NSSI population. In the present study, participants with ongoing NSSI behavior and age‐matched healthy controls conducted a combined OA and OH protocol. The advantage of the combined protocol is that, through a minor adjustment of the thermal stimulation, it allows for the assessment of both endogenous pain inhibition and facilitation. The combined protocol also allows for a comparison of the neural correlates of OA and OH. Our hypotheses were:

1. The NSSI group will display a stronger OA response, compared to healthy individuals
2. The NSSI group will display a weaker OH response, compared to healthy individuals

To our knowledge, this is the first study of OH in a psychiatric population, and the first study combining OH with brain imaging.

## 2 Methods

This experimental study comparing women with NSSI to a control group of healthy women was part of a larger data collection assessing the relationship between NSSI and pain modulation. A description of the characteristics of the NSSI group and the control group can be found in a previously published study (Lalouni et al. 2022). The study was approved by the Regional Ethical Review Board in Stockholm (2018/1367‐31/1). Analysis scripts of the behavioral data can be found at Open Science Framework (https://osf.io/x8hjk). Claude Opus 4.7 (Anthropic) was used for language refinement in selected passages. The authors reviewed all suggestions and take full responsibility for the final manuscript.

### 2.1 Participants

Our power analysis was aimed at detecting differences in pain threshold between NSSI participants and controls. Based on a meta‐analysis by Koenig et al. (2016), we estimated that 29 participants in each group were needed to achieve 80% power. To ensure the ability to detect smaller effects and account for potential incomplete data, we recruited 40 participants for the NSSI group and 40 participants for the control group. We decided to only recruit women because NSSI is more common in women, and we did not think we would be able to recruit the number of male participants to investigate sex differences. fMRI data was collected from 34 out of 40 participants in the NSSI group. Of the six participants for whom fMRI data was not collected, one participant could not remove their piercing; one could not remove their jewelry; one aborted the scan due to claustrophobia; two experienced MR scanner malfunctions; and in one case, the experimenters aborted the scan due to high pain intensity. Three of these six participants completed the experiment outside the scanner, allowing us to collect their pain ratings. As a result, we collected pain ratings from 37 out of 40 participants in the NSSI group. We collected fMRI data and pain ratings from 39 out of 40 participants in the control group. One participant could not complete the experiment because of technical difficulties with the thermal stimulator.

### 2.2 Recruitment

Participants with NSSI and controls were recruited between April 2019 and June 2020, through flyers in waiting rooms at outpatient psychiatric clinics and advertisements in social media. The advertisement for controls was adjusted during the inclusion phase to match the age and educational level of the NSSI participants. General inclusion criteria were: woman; 18–35 years; and right‐handed (to minimize variability in brain lateralization across participants). General exclusion criteria were: chronic inflammatory, autoimmune, or other somatic disorder requiring treatment; pain condition; contraindication for fMRI (e.g., metal implant, pregnancy, claustrophobia); suicide attempts during the last year; suicidal plans or acute risk for suicide. Specific inclusion criteria for participants with NSSI: engaged in self‐injury on ≥ 5 days within the past year. Specific exclusion criteria for controls: treatment for depression or anxiety. Participants with NSSI received a remuneration of approximately $158 (1500 SEK) and controls received approximately $126 (1200 SEK). Participants with NSSI received the higher amount because their participation included a visit to a clinical psychologist (JB) to confirm current NSSI behavior and to assess the risk of participating in the study.

### 2.3 Thermal stimulation and pain assessment

Heat stimuli were administered with a thermal stimulator (Somedic Senselab AB, Hörby, Sverige) with a 30 × 30 mm thermal probe. Temperature increased and decreased at a rate of 5°C/s. Participants used a trackball to rate their pain intensity on a numeric rating scale (NRS) that was displayed on a screen, marked with integers ranging from 0 to 10 on a horizontal line. Participants were instructed verbally that 0 represented “no pain” and 10 “worst imaginable pain.”

### 2.4 Functional MRI Acquisition

Magnetic resonance images were collected with a 3T General Electric 750 MR scanner. We acquired functional scans using T2*‐weighted single‐shot gradient echo planar imaging with the following parameters: repetition time/echo time = 2205/30 ms, flip angle = 80°, field of view = 220 × 220 mm, matrix size = 72 × 72, 42 slices, slice thickness = 3 mm with a 0.5‐mm gap, axial acquisition plane, interleaved slice acquisition. A total number of 220 fMRI volumes were acquired and the total scan time was 8 min. Anatomical images were collected with a high‐resolution BRAVO 3D T1‐weighted image sequence (1 × 1 × 1‐mm voxel size, 176 slices).

### 2.5 Pain Calibration

Pain calibration of the thermal stimulation was conducted on a visit before the experiment. The thermal probe was attached to participants’ left calf. Each thermal stimulation lasted 5 s, with an interstimulus interval of 35 s. Participants used a handheld trackball to rate their pain intensity on a 0–10 NRS that was displayed on a screen after each stimulation. First, starting from 38°C, temperature was raised 1°C for each subsequent stimulation, until the participants rated their pain above NRS 1/10. Second, participants were administered random temperatures, based on their pain threshold. The participants were exposed to between 13–24 heat stimulations (depending on the pain sensitivity of the participant). The temperature never exceeded 50°C, to avoid the risk of tissue damage. Similar to the adaptive thermal pain calibration procedure (Amir et al. 2022), we predicted the temperature closest to NRS 5/10 for each participant using linear regression (temperature ∼ pain rating) based on each participant's pain ratings of the random temperatures.

### 2.6 Experimental Procedure

**FIGURE 1:**
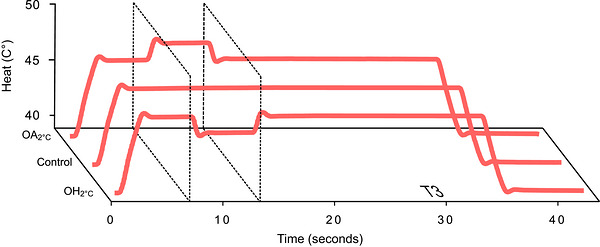
All conditions.

The thermode was attached to the participants' left calf while the participants were lying inside the MRI scanner. Before the start of the experiment, the participants were asked to continuously rate 15 s of thermal stimulation twice. The reason was to confirm the individually calibrated temperature and also familiarize the participants with the rating procedure. The first thermal stimulation was 2°C below the calibrated temperature and the second thermal stimulation was the calibrated temperature. If the maximum pain rating of the calibrated temperature was between NRS 4/10 and 6/10, this temperature was used as the “medium temperature” in the experiment, otherwise the procedure was repeated with a higher or lower temperature (±1°C) until the desired pain rating was reached. The OA and OH protocol can be divided up in 3 time intervals: T1, T2, and T3 (see Figure 1 for a visual representation). During T1 (0–6.5 s), the temperature of the thermode increased from a non‐painful temperature (38°C) to the individual calibrated medium temperature. T1 continued approximately 5 s after the thermode reached the medium temperature. During T2 (6.5–12 s), the temperature was either kept stable (control), increased 2°C (OA), or decreased 2°C (OH) from the medium temperature. T2 continued for 5 s after the thermode reached the assigned temperature. During T3 (12–33 s), the thermode returned to the medium temperature, and after approximately 20 s, the thermode returned to the non‐painful baseline temperature. Pain ratings were continuously registered until approximately 10 s after the thermode returned to the baseline temperature. The three conditions (OA, OH, and control) had the same duration and were repeated during two trials per condition. The order of the trials was randomized for each participant. Every trial was followed by a 50 s inter‐trial interval where the participants were exposed to the baseline temperature of 38°C.

### 2.7 Data Processing and Statistical Analysis

The analysis plan was registered on Open Science Framework on October 25 2021 (https://osf.io/3bquj), after data was collected but before data was analyzed. Statistical signifance was defined as *p*‐values < 0.05 (two‐tailed).

#### 2.7.1 Pain Ratings

**FIGURE 2:**
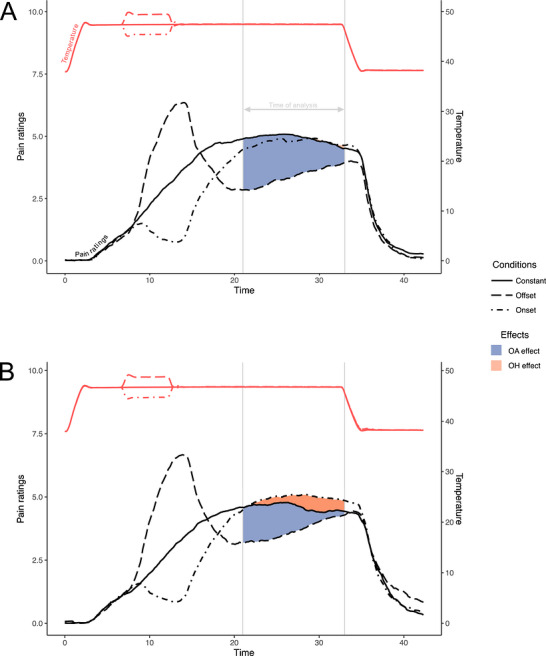
Mean pain ratings and temperature (degrees Celsius) for the NSSI group (A) and control group (B). OA, offset analgesia; OH, onset hyperalgesia; NSSI, Non‐suicidal self‐injury.

First, individual pain ratings were downsampled to 10 Hz to adjust for occasional frame drops (pain ratings were originally collected for each frame rate for the computer screen). Second, we calculated the mean pain rating during the last 12 s of heat stimulation (T3 interval, between 21–33 s of stimulation), for each condition and each participant (Figure 2). This 12‐s interval was decided based on our previous experiments in which this interval produced the strongest OA and OH responses (Fust et al. 2020). Third, we created two mixed effect models using the lme4 and lmerTest packages in R, which allowed us to model the OA and OH responses without averaging the trials. One model estimated the OA response and compared the OA response between groups. The other model estimated the OH response and compared the OH response between groups. In both models, condition, group and the interaction between group and condition were specified as fixed effects. The models also included a by‐subject random effect for condition, specified according to the principle of a maximal random effects structure justified by the design, in order to lower the probability of type I errors and support generalization (Barr et al. 2013). The OA and OH responses were presented as model estimated responses derived from the fixed effects of the mixed effects models.

We also calculated the mean OA and OH response for each participant to test the correlation of the effects. To avoid comparing dependent measures of the OA and OH response by using the same control trials, the mean OA response was defined as the difference between the mean of the pain ratings in the two OA trials and the pain ratings in one of the two control trials (during the last 12 s of heat stimulation), and the mean OH response was defined as the difference between two OH trials and the second control trial. If the OA condition was presented first in the randomized sequence, the two OA trials were paired with the first control trial, and the two OH trials were paired with the second control trial in the randomized sequence, and vice versa. We correlated OA and OH responses across groups (rather than within groups) to maximize statistical power. As an exploratory analysis, we also examined these correlations separately within each group.

#### 2.7.2 fMRI

Quality control of the imaging data was conducted using MRIQC (https://mriqc.readthedocs.io/en/stable/). Preprocessing was performed with the fMRIPrep pipeline (\https://fmriprep.org/), incorporating realignment and spatial normalization to the Montreal Neurological Institute (MNI) standard space. Functional images were subsequently smoothed using an 8‐mm full‐width at half‐maximum (FWHM) Gaussian kernel. Statistical analyses were conducted in SPM12 within MATLAB 2014 (The MathWorks, Inc., Natick, MA). Participants with excessive head movement during the fMRI scan were removed if they had more than 20% of scans with more than 0.5 in framewise displacement during the entire experiment. Two controls and two NSSI participants were removed from the final analysis based on this predefined cutoff.

The experimental design was time‐matched across conditions, such that an equal number of volumes were acquired for the OA, OH, and control condition. Each condition was presented in two blocks. At the first level, OA, OH, and control conditions were modeled as separate regressors in the general linear model (GLM). Six motion parameters (translations in *x*, *y*, and *z* directions and rotations: pitch, roll, and yaw) were included as nuisance regressors. For the group‐level analysis, we used the first‐level contrast estimates for the OA and OH conditions before entering them into the second‐level analysis.

The fMRI BOLD signal for the OA condition across groups was determined on the individual level (first‐level) by subtraction analysis: [OA—Control] resulting in one contrast image per participant that reflected the within‐subject difference between the OA and control conditions. At the group (second) level, these contrast images were entered into a one‐sample *t*‐test to determine whether the mean OA—control effect differed significantly across groups (NSSI and controls together); an approach that accounts for the paired nature of the OA and control conditions at the first level. Next, the interaction group/condition was performed: NSSI [OA—Control] > Healthy controls [OA—Control] using a two‐sample *t*‐test, and the opposite: NSSI [OA—Control] < Healthy controls [OA—Control]. A priori brain regions for region of interest (ROI) analyses for OA include: Brainstem, dorsolateral prefrontal cortex (dlPFC), rostral Anterior Cingulate Cortex (rACC), and thalamus. In addition to the ROI results, we also report exploratory whole‐brain results for the OA responses. The ROIs were based on previous fMRI studies of OA (Derbyshire and Osborn, 2009; Nahman‐Averbuch et al. 2014; Yelle et al. 2009; Zhang et al. 2018). All group analyses were performed using an initial statistical threshold of *p* < 0.001, uncorrected for multiple comparisons, and an extent threshold of 20 voxels. Activation clusters were considered significant at *p* < 0.05, Family Wise Error (FWE) corrected for multiple comparisons.

The fMRI BOLD signal for the OH condition across groups was determined on the individual level (first‐level) by subtraction analysis: [OH—Control], resulting in one contrast image per participant that reflected the within‐subject difference between the OH and control conditions. At the group (second) level, these contrast images were entered into a one‐sample *t*‐test to determine whether the mean OH–control effect differed significantly across groups (NSSI and controls together); an approach that accounts for the paired nature of the OH and control conditions at the first level. Next, the interaction group/condition was performed: NSSI [OH—Control] > Healthy controls [OH—Control] using a two‐sample *t*‐test, and the opposite: NSSI [OH—Control] < Healthy controls [OH—Control]. A priori brain regions for ROI analyses for OH include: S1 and S2, insula, Anterior Cingulate Cortex (ACC), and inferior frontal cortex (IFC). The ROIs were based on brain activation patterns of pain (Jensen et al. 2016). In addition to the ROI results, we also report exploratory whole‐brain results for the OH response. All group analyses were performed using an initial statistical threshold of *p* < 0.001, uncorrected for multiple comparisons, and an extent threshold of 20 voxels. Activation clusters were considered significant at *p* < 0.05, FWE‐corrected for multiple comparisons. All ROIs were based on the Automated anatomical labeling (AAL) atlas 3, with the exception for the brainstem, which was based on meta‐analysis (Linnman et al. 2012).

## 3 Results

### 3.1 Pain Ratings

#### 3.1.1 Offset Analgesia

**FIGURE 3:**
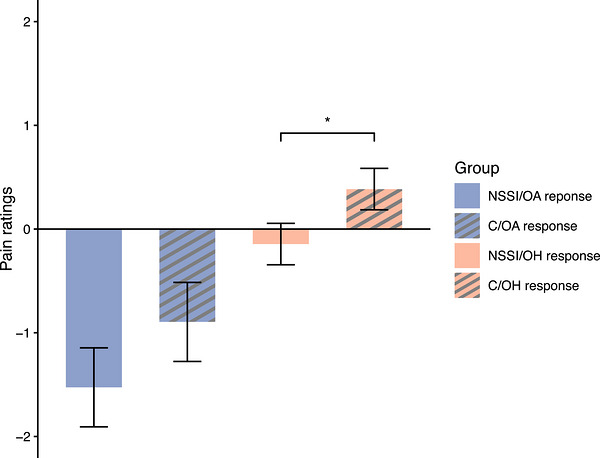
Model estimated OA and OH responses for the NSSI and control group, according to the fixed effects of the mixed effects model. Error bars represent 95% confidence interval of the difference between groups. OA, offset analgesia; OH, onset hyperalgesia; NSSI, Non‐suicidal self‐injury; C, control.

According to the fixed effects of the mixed effects model, model estimated OA response across groups was NRS –0.90 (95% CI –1.43, –0.36, *p* = 0.001; Figures 2 and 3). The model estimated difference in OA response between the NSSI group and control group was not statistically significant (NRS ‐0.63, 95% CI 1.39–0.13, *p* = 0.10; Figures 2 and 3).

#### 3.1.2 Onset Hyperalgesia

According to the fixed effects of the mixed effects model, model estimated OH response across groups was NRS 0.38 (95% CI 0.10–0.66, *p* < 0.001; Figures 2 and 3). The model estimated difference in OH response between the NSSI group and control group was statistically significant (NRS –0.53, 95% CI –0.13, –0.93, *p* < 0.001; Figures 2 and 3). The direction of the result indicates a weaker OH response in the NSSI group compared to controls.

### 3.2 Correlation Between OA Response and OH Response

There was a significant correlation between OA response and OH response (*r* = 0.28; *p* = 0.01) across groups. Exploratory analyses indicated a significant correlation between OA response and OH response in the control group (*r* = 0.30, *p* = 0.01), but not in the NSSI group (*r* = 0.22, *p* = 0.05).

### 3.3 Temperature of the Thermal Stimulation During the Experiment

The mean medium temperature was 45.3°C (SD = 0.6) for the NSSI group and 44.8°C (SD = 1.2) for the control group. For 20 participants in each group, the medium temperature thermal stimulation was set below their calibrated NRS 5/10 for safety reasons.

### 3.4 fMRI

#### 3.4.1 Offset Analgesia

No significant activation clusters related to the OA response were found in either the ROI or exploratory whole‐brain analyses, across or between groups.

#### 3.4.2 Onset Hyperalgesia

**FIGURE 4:**
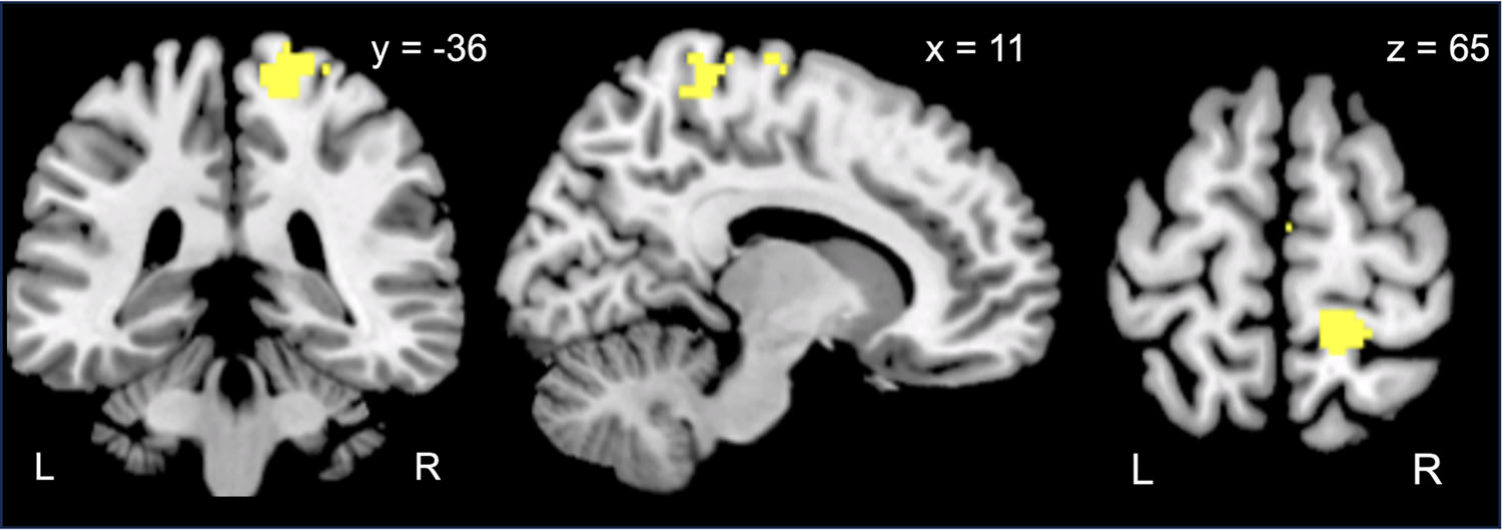
Representation of fMRI BOLD signal associated with the OH response in the primary sensory cortex, compared to the control condition. The result is based on all participants and not a representation of group differences between NSSI and controls. The cluster in yellow is a binary representation of the significant activation contralateral to the pain stimulation. The location of the cluster is indicated by MNI (*x*, *y*, *z*) coordinates. BOLD, blood‐oxygen‐level‐dependent; OH, onset hyperalgesia.

During OH, there was a significant increase of brain activity in the primary somatosensory cortex across groups (Figure 4), approximately corresponding to the contralateral leg area vis a vis the left‐sided pain stimulation on the calf (188 voxels, pFWE = 0.032, MNI coordinate for cluster peak voxel *x* = 14, *y* = –37, *z* = 68). The results suggest an increased activation of the cortical representation of the stimulated leg area during OH. There were no significant clusters in the between‐group comparison, neither in the ROI nor in the whole‐brain analyses. For exploratory reasons, a lowered initial threshold of *p* < 0.05 *uncorrected* of the whole brain indicates that healthy controls had more increases of brain activations during OH, compared to NSSI, represented in primary and secondary somatosensory cortex. Yet, that observation needs to be formally tested in future studies.

## 4 Discussion

In the present study, women with ongoing NSSI behavior and age‐matched control group were tested with a combined OA and OH protocol, during fMRI. Two hypotheses were tested: the NSSI group will display (H1) stronger OA response and (H2) weaker OH response, compared to the control group. The study was part of a larger research project, studying the relationship between NSSI and pain modulation.

In line with the second hypothesis, the women with NSSI behavior displayed weaker OH responses compared to the control group. This suggests that women with NSSI do not facilitate nociceptive signals to the same extent as women without NSSI behavior. The mean difference in pain ratings between groups was small but this was probably due to the low average OH response in this study.

In contrast to the first hypothesis, the OA response was not significantly higher in the NSSI group compared to the control group. The result does not give further support to the notion that the NSSI population has more effective inhibition of pain suggested by some of the earlier studies using conditioned pain modulation, including our own study (Lalouni et al. 2022). Although, it is not clear how other measures of central inhibition of pain, such as conditioned pain modulation, is related to OA. Based on previous studies it is likely that OA does not strongly correlate with conditioned pain modulation (Nahman‐Averbuch et al. 2014). It is possible that conditioned pain modulation is more associated with the hypoalgesic trait that has been observed in the NSSI population than OA.

Across all participants, we registered stronger activation in a region in the primary sensory cortex during the OH condition, compared to the control condition. This was not due to differences in temperature because the temperature was identical in both the OH and control condition during the time of analysis. However, it is still possible that the BOLD signal is related to the afferent nociceptive signal. The nociceptive signal may be upregulated at the spinal and/or peripheral level before reaching the brain. This finding should be interpreted with caution until it has been replicated. It is also important to note that this result is based on both groups and should not be seen as an indication of differences in pain regulation between NSSI and controls. Regarding OA, it has previously been found that the OA response is associated with downregulation of the nociceptive signal in the spinal cord (Sprenger et al. 2018). Even though a null result is technically considered as inconclusive, it is somewhat surprising that we did not find any significant clusters associated with the OA response. Previous studies, including about a quarter to a third of the sample size of this study, reported OA‐related findings in the brainstem (Derbyshire and Osborn, 2009; Nahman‐Averbuch et al. 2014; Yelle et al. 2009) and dorsolateral cortex (Alter et al. 2022; Nahman‐Averbuch et al. 2014; Yelle et al. 2009). There is a need for a large‐scale study on the neural correlates of OA in order to estimate the true effects on pain inhibitory circuitry in the brain.

We found a correlation between OA and OH responses, across all of the participants. A stronger OA response was associated with a weaker OH response, and vice versa. In line with earlier studies (Alter et al. 2020; Fust et al. 2020), this correlation was weak. The weak correlation suggests that OA and OH responses could be related to different pain modulatory mechanisms. Future studies should aim to disentangle the psychophysical properties and biological correlates of OA and OH.

This study has several limitations. First, we compared women with NSSI with a non‐psychiatric control group. This makes it hard to determine if the difference between the groups is attributed to NSSI or other psychiatric traits in the NSSI group. In the NSSI group, approximately half the participants reported a diagnosis of depression, while about a quarter each reported anxiety disorder or borderline personality disorder (Lalouni et al. 2022). Second, there was only a small difference in OH response between the groups. It is possible that this group difference could be affected by other non‐pain related factors such as attention or motivation. Third, for safety reasons, 20 participants in each group received a medium temperature thermal stimulation below their calibrated NRS 5/10. This likely reduced both the OA and OH responses in both groups. Fourth, it is not clear how the temporal relationship between NSSI and pain affected our results. NSSI duration in the NSSI group averaged 10 years, indicating a long history of the behavior (Lalouni et al. 2022). Finally, this study used a slightly different OH protocol (Fust et al. 2020) than the original OH study by Alter et al. (2020). The original OH protocol, which was set at a slightly more noxious temperature during the T1 and T3 intervals, seems to produce stronger and more reliable OH responses and should be used in future studies of OH.

Observational data support the notion that increased pain tolerance constitutes a risk factor for severe NSSI engagement, which may be explained by the role of pain as a barrier to self‐harm (Boyne and Hamza, 2024). If it can be established that individuals who engage in NSSI behavior exhibit altered pain modulation, this could at least partially account for the elevated pain tolerance observed in this population. Evidence of hyper‐effective pain modulation in the NSSI would also provide a potential target for pharmacological interventions increasing the sensitivity to pain, such as opioid antagonists. There was a range of OA and OH responses in both the NSSI and the control group, as can be expected in psychophysical pain measures in any population (Petersen et al., 2025; Vincenot et al. 2026). With a larger sample size it would likely be possible to identify subgroups within these groups, which may also vary in terms of responses to different types of treatment.

To our knowledge, this is the first study using OH to investigate pain modulation in a psychiatric patient group. To assess whether OH is a clinically relevant measure of endogenous pain modulation, it would be valuable to compare a patient group with a pronociceptive pain modulation profile, such as patients with nociplastic pain with a group of healthy individuals. Detailed information on pain modulation in the NSSI population and patients with nociplastic pain, in combination with their psychiatric comorbidity, might be key to understanding how individuals move along the nociceptive spectrum.

## Author Contributions

**Jens Fust**: conceptualization, investigation, writing – original draft, methodology, visualization, writing – review and editing, formal analysis. **Granit Kastrati**: Writing – review and editing, formal analysis. **Johan Bjureberg**: investigation, writing – review and editing, methodology. **Nitya Jayaram‐lindström**: writing – review and editing, methodology. **Clara Hellner**: methodology, writing – review and editing. **Eva Kosek**: methodology, writing – review and editing. **Peter Fransson**: methodology, writing – review and editing. **Karin B. Jensen**: conceptualization, methodology, writing – review and editing, formal analysis, supervision, writing – original draft, funding acquisition. **Maria Lalouni**: supervision, conceptualization, investigation, writing – original draft, writing – review and editing, methodology, formal analysis.

## Funding

This work was supported by grants from the Swedish Research Council (2019‐02936), StratNeuro Collaborative Grant, and a donation from Leif Lundblad to pain research. Open access funding is provided by Karolinska Institutet.

## Ethics Statement

The study was approved by the Regional Ethical Review Board in Stockholm (2018/1367‐31/1). We agree to Wiley's Best Practice Guidelines on Research Integrity and Publishing Ethics.

## Patient Consent Statement

All participants signed a written consent to participate in the study.

## Conflicts of Interest

Eva Kosek reports personal fees from UCB Pharma, Orion and Eli Lilly outside the submitted work. The other authors declare no conflicts of interest.

## Data Availability

Data is available upon reasonable request
